# Metabolic and Physiological Responses of Shiraz and Cabernet Sauvignon (*Vitis vinifera* L*.*) to Near Optimal Temperatures of 25 and 35 °C

**DOI:** 10.3390/ijms161024276

**Published:** 2015-10-14

**Authors:** Uri Hochberg, Albert Batushansky, Asfaw Degu, Shimon Rachmilevitch, Aaron Fait

**Affiliations:** 1 Department of Agricultural and Environmental Sciences, University of Udine, via delle Scienze 208, 33100 Udine, Italy; E-Mail: uriho9842@yahoo.com; 2 Albert Katz International School, Ben Gurion University of the Negev, 84990 Sede Boqer, Israel; E-Mails: albert.batushansky@gmail.com (A.B.); degua3@gmail.com (A.D.); 3 The French Associates Institute for Agriculture and Biotechnology of Drylands (FAAB), the Jacob Blaustein Institute for Desert Research, Ben Gurion University of the Negev, 84990 Sede Boqer, Israel; E-Mail: rshimon@bgu.ac.il

**Keywords:** grapevine, *Vitis vinifera*, heat, temperature, metabolite profiling, metabolism, plant physiology, network analysis

## Abstract

Shiraz and Cabernet Sauvignon (Cs) grapevines were grown at near optimal temperatures (25 or 35 °C). Gas exchange, fluorescence, metabolic profiling and correlation based network analysis were used to characterize leaf physiology. When grown at 25 °C, the growth rate and photosynthesis of both cultivars were similar. At 35 °C Shiraz showed increased respiration, non-photochemical quenching and reductions of photosynthesis and growth. In contrast, Cs maintained relatively stable photosynthetic activity and growth regardless of the condition. In both cultivars, growth at 35 °C resulted in accumulations of secondary sugars (raffinose, fucose and ribulose) and reduction of primary sugars concentration (glucose, fructose and sucrose), more noticeably in Shiraz than Cs. In spite of similar patterns of metabolic changes in response to growth at 35 °C, significant differences in important leaf antioxidants and antioxidant precursors (DHA/ascorbate, quinates, cathechins) characterized the cultivar response. Correlation analysis reinforced Shiraz sensitivity to the 35 °C, showing higher number of newly formed edges at 35 °C and higher modularity in Shiraz as compared to Cs. The results suggest that the optimal growth temperatures of grapevines are cultivar dependent, and allow a first insight into the variability of the metabolic responses of grapevines under varied temperatures.

## 1. Introduction

Most common grape cultivars were propagated in a wide variety of climatic regions [1], and as such, they adapted to equally varied environmental conditions, including temperature [2]. Over the past few centuries, however, grape varieties have been bred to grow well in temperate environments. With the recent increases in wine demand in regions not traditionally associated with its consumption, such as the Middle East and South-East Asia, grape cultivars are grown in climates that differ markedly from those where they originated. The widespread cultivation of grapes in warmer areas and global climate change processes altered the temperatures of most grape growing areas in the past 55 years [3] and exposed them to frequent heat wave events. By the year 2050, the annual average temperature in grape-growing regions is projected to increase by 0.4 to 2.6 °C [2]. Studies have focused on the effect of increased temperatures on fruit and wine quality [4,5,6,7,8], examined grape leaf stress physiology [9,10,11], summarized the general response of grape leaves to varying temperatures [12], observed the anatomy of grapevines under heat stress [13], and recently investigated leaf antioxidants’ metabolism in respect to different cultivars [14]. Metabolic profiling of grapevine leaves was not yet measured and should provide a wider perspective of grapevine introduction to warmer climates.

The performances of grapevine, a C3 plant, decline under heat stress leading to a potential damage to yield and fruit quality [10]. However, when considering the optimal growth temperature for grapes, studies have suggested a wide range of temperatures (25–35 °C [11,12]), which may depend on plant developmental stage [15] and on the cultivar itself [16,17]. The common response of C3 plants to increased temperatures includes the reduction of net photosynthesis and increased respiration [18,19]. Additionally, non-photochemical quenching (NPQ) may serve as a good indicator for limitation in the photosynthetic apparatus as it dissipates the excessive energy as heat [11]. Since the above mentioned parameters are relatively easy to measure with commercial gas exchange–fluorescence systems, they commonly serve as good heat limitation indices.

Environmental stresses are complex stresses characterized by interconnectedness of environmental factors. Radiation, humidity, water availability, salinity, and temperature are connected through the heat balance [20], in which radiation is the energy source and water is part of the energy sink. This interconnectedness limits the ability to characterize plant response based on any single environmental factor. For example, an increase in temperatures will likely increase vapor pressure deficit (VPD), evapotranspiration and salinity while reducing the availability of water. Through VPD management, the effect on environmental factors—other than temperature—could be diminished. The present study aimed at deepening our understanding of heat response in a VPD-controlled environment by comparing leaf physiology of two grapevine cultivars.

Recently, we explored the metabolic response to drought of Shiraz and Cabernet Sauvignon (Cs) identifying the milder perturbation of Cs metabolism as compared with Shiraz [21,22,23]. Here, we describe a physiologic and metabolic investigation of the varietal response to different temperatures and heat stress. Shiraz and Cs grapevines were exposed to two temperature treatments of 25 and 35 °C, for seven days. Leaf photosynthetic parameters and metabolite profiles were investigated under the different conditions and network analysis was employed to explore the cultivars metabolic coordination under varied temperatures.

## 2. Results

Differences in plant physiology and metabolites among the measuring days were insignificant. Similarly, Guy *et al.* [24] showed that the majority of the metabolite responses to high temperature occurred within the first 30 min. After validation of the normal distribution of the data (Shapiro Wilk test), data from the second, fourth, and sixth days were bulked and both experiments were averaged.

**Figure 1:**
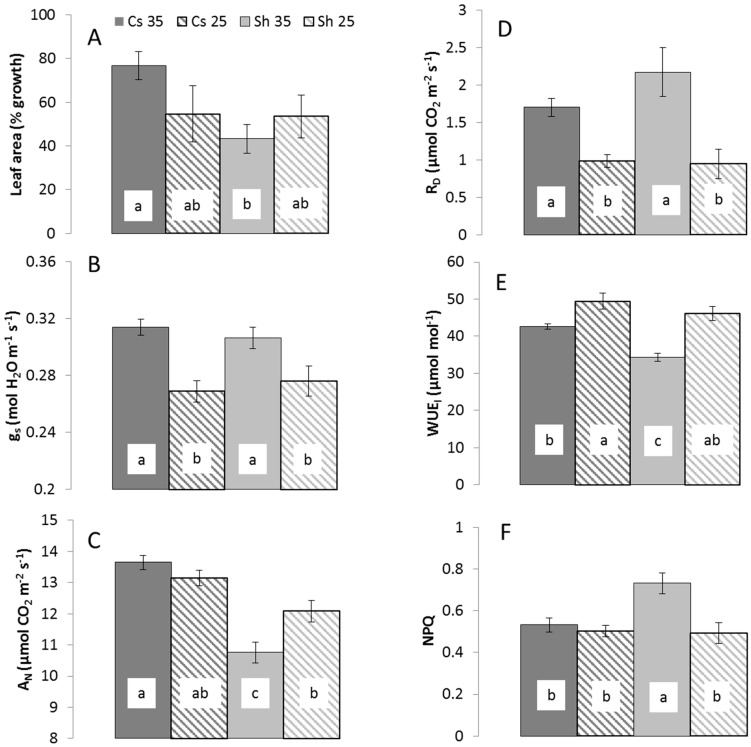
Leaf area growth as a percent of the leaf area in day 1 (**A**) Stomata conductance (*g*_s_; (**B**)) net assimilation (*A*_N_; (**C**)) dark respiration (*R*_D_; (**D**)) intrinsic water use efficiency (WUEi, (**E**)), and non-photochemical quenching (NPQ; (**F**)) of Shiraz and Cs on the 25 and 35 °C treatment. Data are means ± S.D of all measuring days of the two experiments. *n* = 8. Different letters represent significant difference between treatments.

Results showed similar values in both cultivars in most physiological parameter under 25 °C, but signs of stress were noticed under 35 °C only in Shiraz. The growth (expressed by additional leaf area) measured for Shiraz and Cabernet Sauvignon (Cs) cultivars after six days of exposure to 25 °C were similar ~54%. (Figure 1A). However, when exposed to the 35 °C temperature for six days, the growth of Shiraz (43%) was lower than that of Cs (77%, Figure 1A).

Throughout both experiments, the stomata conductance (*g*_s_) of both cultivars was similar. It was ~0.27 mol H_2_O m^−2^·s^−1^ in the 25 °C treatment and significantly higher (~0.34 mol H_2_O) in the 35 °C (Figure 1B). The net assimilation rates (*A*_N_) at 25 °C were 12.1 and 13.1 µmol CO_2_ m^−2^·s^−1^ for Shiraz and Cs, respectively (Figure 1B). At 35 °C, the *A*_N_ of Shiraz was significantly reduced to 10.75 µmol CO_2_ m^−2^·s^−1^ (Figure 1B) but that of Cs was not affected. Additionally, at 35 °C, other stress indicators such as dark respiration (*R*_D_) (2.2 and 1.7 µmol CO_2_ m^−2^·s^−1^ for Shiraz and Cs, respectively) and WUEi were more noticeable in Shiraz (Figure 1D,E). Last, non-photochemical quenching (NPQ) values, an important heat stress physiological response, markedly changed only in Shiraz (from 0.5 at 25 °C to 0.7 at 35 °C), suggesting that the reduction in the energy tunnelled to photochemistry, likely dissipated as heat (Figure 1F).

### 2.1. Metabolic Response to Temperature

**Figure 2:**
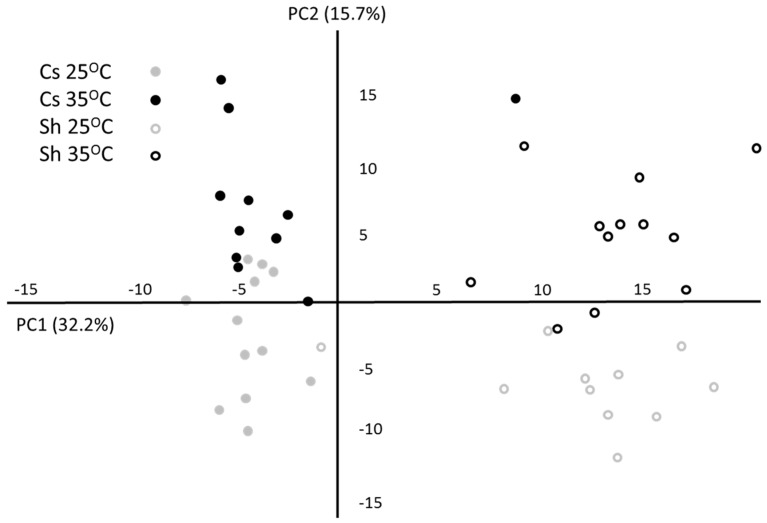
Principal component analysis (PCA) plot (*x*—1st component; *y*—2nd component) of metabolite profiling data from Cabernet Sauvignon (Cs) (full) and Shiraz (Sh) (empty) samples grown at 25 °C (grey) and 35 °C (black) treatments.

A total of 64 leaf metabolites were identified, of which 36 were significantly different between growth temperatures in at least one of the cultivars (Table S1). The metabolic alteration was slightly larger in Shiraz in number of significantly changed metabolites (28 as compared with 24 in Cs) and in the extent of their average change in relative abundance (53% as compared with 47% in Cs). Metabolite profile based principal component analysis (PCA) was used to group treatments and cultivars (Figure 2). Explaining 32.3% and 15.7% of data variance, the first and second components separated between cultivars and between the temperature treatments, respectively. The eigenvalues of the first component (cultivar) were characterized mainly by quinate 5 caffeoyl, quinate, glucuronate and threonate, which together contributed the most to the separation based on cultivar metabolic profile (Table S2). The eigenvalues of the second component (temperature treatment) included the polyamine putrescine, and major carbon metabolites: glucose, malate and raffinose.

Comparing carbon metabolism under the two temperature regimes revealed many other notable changes (Table 1). Citrate was increased by 39% in Cs and 64% in Shiraz when plants were grown at 35 °C compared with plants grown at 25 °C. Other detected TCA (tricarboxylic acid) cycle intermediates such as succinate, and malate were reduced by 52% and 64% in Cs, respectively, and by 485%, and 43% in Shiraz, respectively, in plants grown at 35 °C compared with those grown at 25 °C. Sugar metabolism was significantly affected by growth temperature (Table 1), more notably in Shiraz than in Cs. The major plant sugars, including glucose, fructose and sucrose, were reduced in abundance when vines were grown at 35 °C compared with 25 °C in Cs (by 39%, 10% and 33%, respectively) and in Shiraz (by 61%, 43% and 33%, respectively). In contrast, at 35 °C fucose, ribulose, erythronate, and raffinose increased 52%, 70%, 53% and 81%, respectively, in Cs and 84%, 44%, 108% and 213%, respectively, in Shiraz compared with growth at 25 °C.

Increased temperature also caused a decreased abundance of dehydroascorbate (DHA), shikimate and glutarate by 15% to 30% in both cultivars. In contrast, all other detected intermediates of the carbon metabolism, including ascorbate, galactonate, malonate, and phosphorate, were increased by 20% to 80% in the plants grown at 35 °C. As a consequence, the ratio of ascorbate to DHA, known for its importance in the scavenging of reactive oxygen species (ROS), increased from 0.78 (at 25 °C) to 1.25 (at 35 °C ) and from 0.9 (at 25 °C) to 1.56 (at 35 °C) in Shiraz and Cs, respectively.

**Table 1:** Ratio of relative metabolite abundance at 35 and 25 °C in Shiraz (Sh) and in Cabernet Sauvignon (Cs). Only metabolites that were significantly different between the treatments in at least one of the cultivars are presented. Values are presented as fold change of mean metabolic abundance (*n* = 12) in the 35 °C treated samples compared with the 25 °C treated samples. Bolded values represent significant (*p* < 0.05) differences between the treatments.

Metabolite	Cs 35/25	Sh 35/25
Amino acids		
Ala	**1.38**	**1.60**
Gly	**0.66**	**0.69**
Ser	**0.41**	**0.54**
Glu	**0.47**	0.64
Asp	1.67	**1.71**
Organic acids		
Citrate	1.39	**1.64**
Malate	**0.36**	**0.57**
Maleate	**0.72**	**0.77**
Succinate	**0.48**	**0.52**
Threonate	0.67	**0.55**
Threonate-1,4-lactone	**0.71**	0.74
Glycerate	**0.56**	**0.51**
Malonate	**1.26**	1.19
Phosphorate	1.25	**1.53**
Erythronate	**1.53**	**2.08**
Glutarate 2-oxo	**0.71**	0.96
Arabinonic acid	1.38	**1.61**
4-Hydroxy *trans*-cinnamic acid	**1.79**	**1.52**
4-Hydroxy *cis*-cinnamic acid	**1.27**	1.11
Shikimate	0.74	0.85
Dehydroascorbate	**0.74**	**0.71**
Ascorbate	**1.28**	1.15
Glucuronate	**1.36**	**1.45**
*cis*-Caffeaet	**1.28**	1.16
Galactonate	**1.54**	**1.64**
*trans*-Caffeate	**1.32**	1.09
Sugars		
Fructose	0.90	**0.53**
Glucose	**0.61**	**0.40**
Glucose-6-phosphate	**0.62**	**0.62**
Sucrose	**0.68**	**0.63**
Xylose	0.84	**0.82**
Fucose	**1.53**	**1.82**
Ribulose	**1.70**	**1.44**
Raffinose	**1.81**	**3.13**
Others		
Putrescine	**0.28**	**0.34**
Epicatechin	**0.55**	0.85
Catechin	0.79	**1.37**
Epigallocatechin	0.99	**1.57**

Cultivar growth at the higher temperature caused a significant decrease in the respiration related amino acids: Gly, Ser, and Glu of 34%, 60% and 53%, respectively, in Cs and of 31%, 44% and 47%, respectively, in Shiraz (Table 1). In contrast, Ala and Asp were higher in abundance by 37% and 66%, respectively, in Cs and by 60% and 71%, respectively, in Shiraz at 35 °C *vs.* 25 °C. Plants grown at 35 °C were also characterized by significantly reduced amounts of the polyamine putrescine, namely, 72% and 64% less in Cs and Shiraz, respectively. The identified flavonoids catechin and epigallocatechin were elevated at 35 °C by 36% and 57%, respectively, in Shiraz compared to their levels at 25 °C, but, in Cs, these two catechins were not affected by growth temperature. In contrast, epicatechin was 45% lower at 35 °C than at 25 °C in Cs, but in Shiraz it was unchanged.

### 2.2. Cultivar Metabolic Differences

Exploring the differences between cultivars in relative metabolite abundance (Table S3) resulted in the identification of 18 differential metabolites at 25 °C and 16 at 35 °C (Table 2). The majority of the metabolites detected had a similar ratio between Cs and Shiraz, regardless of the treatment (Table 2). The detected *N*-compounds that were substantially different between the cultivars were butanoate 2,4-dihydroxy (~35% higher in Cs) and Glu (25%–45% higher in Cs). Significant differences between the two cultivars (10%–50%) were also found among many of the organic acids, of which quinates had the largest difference. Namely, quinate was 1.7 and 3 times higher in Shiraz than in Cs at 35 and 25 °C respectively, while quinate 5 caffeoyl was 34 and 14 times higher in Cs than in Shiraz at 35 and 25 °C, respectively. Most sugars (glucose, xylose, lyxose, raffinose) were 15%–45% lower in Cs than in Shiraz, but ribulose was 91% and 61% higher in Cs under the 35 and 25 °C treatments, respectively. Flavonoid abundance was also cultivar dependent. Catechin and epicatechin were twice as abundant in Shiraz at 25 °C but were similar in both cultivars at 35 °C. However, epigallocatechin was 1.5 (35 °C) and 2.4 (25 °C) times higher in Cs.

**Figure 3:**
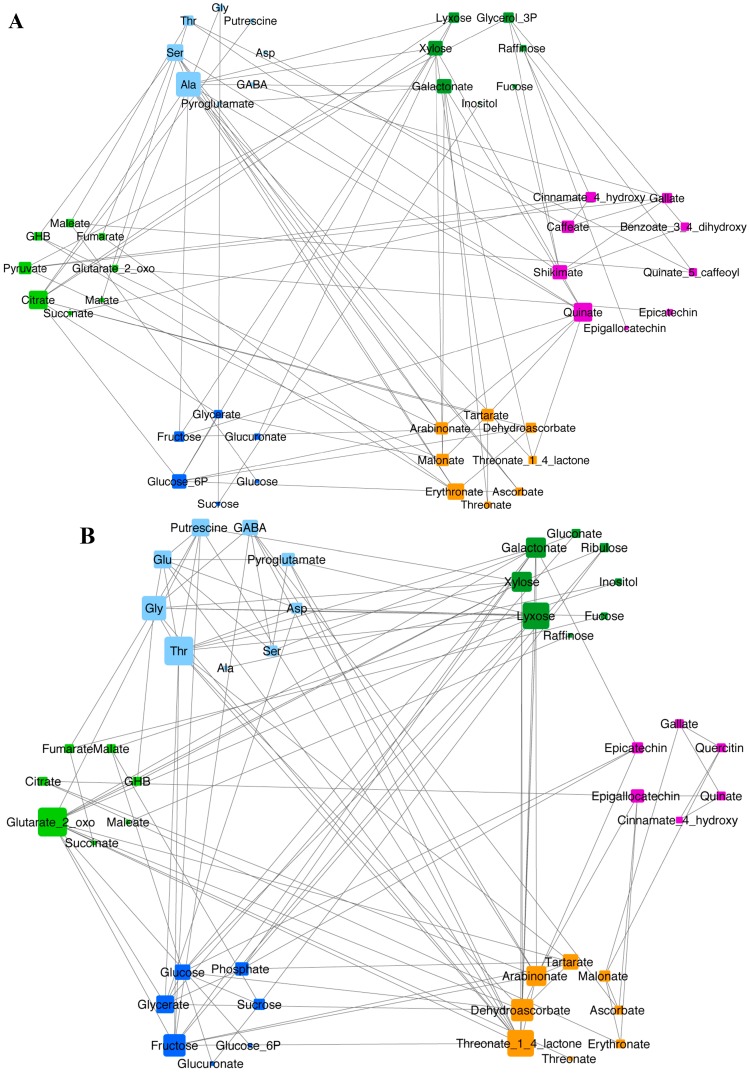
The metabolic networks of Cs (**A**) and Sh (**B**) varieties built based on Spearman’s rank correlation. Each node represents a metabolite, each edge—significant correlation between pairs of metabolites, unique for each variety. The size of nodes represent nodal degree from 1—the smallest to 11 (**A**) or 12 (**B**)—the largest. The color code of the nodes represent the metabolic pathway affiliation: amino acid metabolism (blue), glycolysis (navy), TCA cycle (light green), non-glycolytic sugars (dark green), flavonoids pathways (purple) and carboxylic acids (orange).

To investigate the metabolic coordination of the cultivar response to increased temperature of growth, correlations analysis was performed and visualized (Figure 3). Generally, most of the properties of the two networks were similar (Table 3). The proportion of newly formed edges in the networks (80% for Shiraz and 75% for Cs) suggests a strong effect of growth at 35 °C on both varieties. Nevertheless, the larger number of 35 °C-specific edges in Shiraz compared to Cs (109 *vs*. 84) indicates a broader response of this variety to the imposed conditions. Analysis of the network properties, showed increased modularity in the Shiraz network based on biochemical pathways segregation. The observed co-response of the main metabolic routes can be seen in Figure 3. A similar range of the nodal degree was measured within the two networks. Cultivar differences in the relations between biochemical classes were observed. For example, between amino (blue) and carboxylic (orange) acids connections are mostly via Ala, Ser and Threonate in Cs (Figure 3A). In contrast, in Shiraz, a stronger relationship between these two classes is observed which involves Thr, GABA, 2-oxo-glutarate, pyroglutamate, threonine-lactone, ascorbate, dehydroascorbate, tartarate (Figure 3B). Similarly, increased number of connected nodes in Shiraz characterizes also the relationships between amino acids and glycolytic sugars, amino acids and non-glycolytic sugars, and glycolytic and non-glycolytic sugars. Notably, the involvement of flavonoids in the Cs network is much stronger compared to Shiraz (Figure 3).

**Table 2:** Comparison of metabolites of Shiraz (Sh) *vs.* Cabernet Sauvignon (Cs) in the 35 and 25 °C treatments. Only metabolites that were significantly different between the cultivars in at least one of the treatments are presented. Values are the fold change of mean metabolic content (*n* = 12) in Cs compared with in Sh. Bolded values represent significant differences between the treatments.

Metabolite	Cs/Sh 35	Cs/Sh 25
Amino acids		
Butanoate 2,4-dihydroxy	**1.34**	**1.38**
Glu	1.25	**1.45**
Organic acid		
Maleate	**0.63**	**0.67**
Malate	**0.32**	**0.50**
Phosphorate	1.23	**1.51**
Glutarate	**0.81**	1.11
4-hydroxy *cis*-cinnamic acid	**1.43**	1.26
Quinate	0.58	**0.33**
5-caffeoyl *trans*-quinate	**33.83**	**13.65**
Threonate	**0.45**	**0.37**
Ascorbate	**1.24**	1.11
Glucuronate	**0.52**	**0.41**
Galactonate	**0.70**	**0.75**
Caffeate	0.99	**0.82**
Others		
catechin	**0.50**	0.87
Epigallocatechin	**1.49**	**2.37**
Epicatechin	**0.60**	0.93
Sugars		
Glucose	**0.71**	**0.68**
Raffinose	**0.56**	0.97
Xylose	**0.74**	**0.72**
Lyxose	0.84	**0.81**
Ribulose	**1.92**	**1.61**

**Table 3:** The main Graph theory based properties calculated for the correlation-based networks of Cabernet Sauvignon (Cs) and Shiraz (Sh) varieties.

Variety	# of Nodes	# of Edges	Common Edges	Ø	Transitivity	Modularity		
						Biochemical	Walk-Trap	Edge Betweenness
Cs	48	112	28 (25%)	10	0.4	6 (−0.01)	9 (0.5)	7 (0.5)
Sh	48	137	28 (20%)	9	0.5	6 (0.1)	11 (0.4)	11 (0.4)

## 3. Discussion

The present study tested grapevine varietal differences of physiology and metabolism in response to near optimal temperatures. Our results suggest that Shiraz was more sensitive to growth at 35 °C than Cs. This was evident by lower growth rate, reduced assimilation rate (*A*_N_), increased respiration (*R*_D_), increased non-photochemical quenching (NPQ), lower intrinsic water use efficiency (WUE_i_), higher metabolic shift—both in number and extent of change in the many metabolites measured, and higher alteration of several metabolic stress indicators such as raffinose and sucrose. In accordance with that, correlation based network analysis revealed a smaller network diameter in Shiraz, reflecting a swifter response of the system to stress as previously suggested [25].

Cultivar physiology was similar under the 25 °C treatment, but differed significantly at 35 °C. Overall, Shiraz displayed significantly lower leaf growth than Cs at 35 °C, which can be explained by its lower *A*_N_ rates. Higher *R*_D_ rates in Shiraz coincided with its lower levels of primary sugars, also suggesting a limited carbon assimilation at 35 °C. Our results imply that the substantial varietal diversity documented in the photosynthetic performances of different grapevine cultivars [26,27] may be the result of differential cultivar responses to temperatures and could be either diminished or augmented under different temperature regimes. Modelling of grapevines behaviour in a changing climate [2,3,12,28,29] should address cultivar differences. The extent of the predicted effects depends on the cultivar and will probably be higher in Shiraz than in Cs.

Combining and comparing metabolite profiling with physiological parameters is a non-trivial task, but can provide higher resolution of leaf cell physiology and stress response [30,31]. For instance, Cs photosynthesis and growth at 35 °C was not impaired, and even improved compared with the 25 °C conditions. It is thus difficult to determine if the modifications in primary metabolism (similar in both cultivars) are indicative of stress. It appears that the overall response in number of significantly changed metabolites and the average degree of change (both higher in Shiraz) correlates well with the alteration in photosynthetic parameters, but less indicative of impaired growth. Similar observations were previously made in leaf and berry skin metabolism of the same cultivars in response to water stress [21,23]. These studies showed a link between the degree of primary metabolism modification and the stress degree, while qualitative differences between the cultivars were rare. Overall, these findings emphasise the challenging task of characterizing a metabolic profile indicative either of stress or tolerance and highlight the importance of combining supportive physiological measurements.

Alteration of TCA intermediates at higher growth temperatures was in line with almost double *R*_D_ values in both cultivars. The latter phenomenon was expected as a probable outcome of a 10 °C increase in measurement temperature (*i.e.*, *Q*_10_ = 2), although this assumption is often incorrect [18]. Comparisons of the patterns of change in TCA intermediates in grapevine and other plant species under elevated temperatures [32,33] revealed a common characteristic increase in citrate content. However, the lower relative abundance of malate and succinate observed in our study seems specific to grapevines [32,33] and is suggestive of significant cataplerotic activity of the TCA cycle coupled to a likely oxidative impaired 2 oxoglutarate dehydrogenase activity [34]. This phenomenon, more noticeable in Shiraz than in Cs, provides additional support for the greater sensitivity of Shiraz to the 35 °C conditions.

Sugars are known to be of critical importance to plant responses to high temperatures and to other abiotic stresses [35,36]. It has long been known that grapevine responses to heat significantly affect sugar content [37] and the sugar partitioning exhibited by grapevines under water stress was suggested to be unique among species. In most plants, grapevines included [38], high temperatures result in breakdown of starch into soluble sugars that provide substrates for increased respiration [39]. In previously studied plants—creeping bentgrass (*Agrostis stolonifera*) [40], rice (*Oryza sativa*) [41], grasses (*Cynodon transvaalensis* × *Cynodon dactylon* (C_4_) and *Poa pratensis* (C_3_)) [33] and *Arabidopsis thaliana* [32,42]—the exposure to high temperatures elicited an increase in soluble sugars. However, the results of this study (Table 1) showed a decrease in the abundance of primary soluble sugars (glucose, fructose, sucrose), and an increase of other sugars (ribulose, fucose, erythronate, raffinose) in response to the 35 °C treatment. Similar results were found by [38] in response to heat stress and by several other studies in response to different environmental stresses [30,43,44,45]. The opposite response of primary (decrease) and secondary (increase) sugars at higher temperatures might reflect a metabolic consequence of the reduced photosynthesis and the activation of stress related metabolism (e.g., raffinose metabolism) [32,33].

The occurrence of a stress condition evolving at 35 °C was reflected by the plants’ altered antioxidant metabolism [46], as shown by changes in the abundance of epigallocatechin, a hydrophobic flavonoid that can be integrated into lipid bilayers and protect membrane proteins and lipids from oxidative damage [47], and by the alteration of ascorbate metabolism. A high ratio of reduced to oxidized ascorbate is essential for ROS scavenging in cells [46] and in plant cell responses to heat shock [48]. The significantly higher basal levels of both antioxidants in Cs possibly contributed to sustain heat induced oxidative stress, leading to its higher assimilation rates at 35 °C. The importance of antioxidants activity in grapevine acclimation and adaptation to heat was recently highlighted [14] and associated with differential performances of Touriga Nacional and Trincadeira grapevine cultivars. The strongest metabolic response to the 35 °C condition in both cultivars was a significant reduction in stress related polyamine putrescine, which was found to regulate abscisic acid (ABA) levels [49]. Moreover, low levels of this polyamine can reduce the ratio of electric to osmotic components of the proton motif force in the thylakoids [50], which could eventually lead to the increased NPQ measured at 35 °C.

Increased temperatures frequently lead to high VPD. In turn, VPD affects grapevine physiology, either directly or via other related environmental conditions (mostly water availability; [51,52]). As expensive facilities are required to manipulate temperatures without affecting VPD, their combined effect is normally studied even though some physiological mechanisms have contrasting responses to these conditions. For example, high temperatures are commonly responded by stomatal opening for cooling purposes [53], but the accompanied high VPD leads to stomatal closure [54]. Accordingly, in grapevine studies, heat stress led to either an increase [11,16,55] or a reduction (Figure 1, [8]) of *g*_s_, possibly signifying the dominance of either heat or VPD on stomata regulation at that specific study. The differences between the above mentioned studies demonstrate the potential confusion when drawing conclusion from the combined effect of heat and VPD. In the present study, common metabolic responses to water stress, such as sugars and amino acid accumulation [30,43,44], were not found, though previous grapevine heat studies found their response [32,42]. The genotypic and environmental (other than VPD) diversities between the studies may be the sources of these differences. Nonetheless, the effect of VPD on plant physiology in temperature manipulation experiments should be further investigated.

Network differences between Shiraz and Cs are similar to our previous findings under water stress conditions [23]. In both studies, stress conditions resulted in larger networks, but relatively moderate changes in metabolite correlations characterized Cs, a further indication of its resilience to the 35 °C. Positive modularity based on biochemical pathways in Shiraz in response to 35 °C indicates a greater coordination of changes in the main central metabolic routes.

To conclude, significant changes in physiology, metabolite relative abundance and the metabolic network topology characterize a grapevine response to temperature modification within the optimal range (25–35 °C). Additionally, the study shows that optimal growth conditions are cultivar dependent; namely, Shiraz was shown to be more sensitive to 35 °C temperatures physiologically and metabolically and will likely exhibit a stronger response to the higher temperatures predicted for the near future.

## 4. Materials and Methods

### 4.1. Plant Material

Two-year-old *Vitis vinifera* cv. Shiraz 174 and Cabernet Sauvignon 169 (Cs) grafted on Richter 110 were planted in 9-L plastic pots filled with 8 L of potting media (RAM8; Tuff Merom Golan, Merom Golan, Israel) that included a slow-release fertilizer (Elgo, Multigan, Israel). Prior to the experiment, plants were grown in a greenhouse and irrigated daily to field capacity. Three weeks prior to the experiments, plants were pruned to two-bud spurs and were regrown to a single shoot of about 70 cm. Before beginning the heat experiment, both cultivars were acclimated for three days in the growth chambers with day/night cycles of 25/20 °C.

### 4.2. Treatments and Experimental Designs

Vine responses to different temperatures were tested using two growth chambers for a period of seven days (Percival Scientific Inc., Perry, IA, USA). Due to space limitation in the growth chamber (support only eight vines/growth chamber) the experiment was repeated in order to increase the significance of the results. In each experiment, plants (eight Shiraz and eight Cs plants) were grown for seven days in one of two growth chambers (four Shiraz and four Cs plants in each chamber). For the first six days, one chamber had day/night temperatures of 35/30 °C and the second chamber was kept at 25/20 °C, reflecting the temperature range (25–35 °C) traditionally considered to be optimal for grape leaf photosynthesis [11,12]. In both cultivars leaf temperature, measured using a thermal camera (FLIR systems), was 32.7 ± 0.3 °C in the 35 °C chamber and 24.7 ± 0.3 °C in the 25 °C chamber. Day/night cycles consisted of 16/8 h with daytime light intensity of 500 μmol m^−2^·s^−1^. To prevent the results from being metabolically and physiologically skewed by differential VPD values, the humidity of each chamber was controlled by built-in the (de)-humidifier system to ensure that a VPD of 0.855 kPa was maintained, regardless of temperature. To ensure similarity of VPD in the two growth chambers, four 50 mL tubes filled with water were placed in each chamber and weighed periodically during the experiment. No significant difference was found (*p* = 0.42) between the evaporation in the two chambers. During the experiment, all plants were watered regularly as described during their establishment period.

### 4.3. Leaf Area

Leaf area was determined using a linear model (Area = 0.3281 × length^2.55^ (*R*^2^ = 0.9)) that transformed leaf length to area [56]. On the first and seventh days of the experiment, all the leaves of each plant were measured and their areas were calculated. Leaves that were sampled during the experiment were measured and taken into consideration.

### 4.4. Gas Exchange and Chlorophyll Fluorescence Analysis

In each of the two experiments, photosynthetic parameters were measured on the second, fourth, and sixth days. The LiCor-6400 portable photosynthesis system (Licor, Lincoln, NE, USA) was used to measure gas exchange and chlorophyll fluorescence parameters. From each plant a young fully mature leaf was measured under a constant light intensity of 500 μmol·m^−2^·s^−1^ and a CO_2_ concentration of 400 μmol·mol^−1^. The gas exchange parameters recorded were net carbon assimilation (*A*_N_), stomata conductance (*g*_s_), and dark respiration (*R*_D_). Non photo-chemical quenching (NPQ) was measured exactly as described in [57].

### 4.5. Leaf Sampling and Extraction for Metabolite Profiling

Mature, fully expanded leaves were sampled from each plant on the second, fourth, and sixth days of the experiment for metabolic analysis. Sampling, storage and extraction for metabolic analysis were performed exactly as previously described [23]. On all sampling dates, leaf samples were collected, immediately snap frozen with liquid nitrogen, and then kept at −80 °C until further analysis. Samples for GC–MS analysis were processed following the method of [58]. After extraction, 100 µL of the water/methanol phase was dried in a vacuum concentrator (Eppendorf Concentrator Plus) for derivatization [59] for GC–MS analysis.

### 4.6. GC–MS Derivatization and Data Processing

Samples for GC–MS analysis were processed as was previously described [23]. Residues were redissolved and derivatized for 120 min at 37 °C (in 40 µL of 20-mg/mL methoxyamine hydrochloride in pyridine) followed by a 30-min treatment with 70 µL *N*-methyl-*N*-(trimethylsilyl) trifluoroacetamide at 37 °C. Eight microliters of a retention time standard mixture (0.029% *v*/*v* *n*-dodecane, *n*-pentadecane, *n*-nonadecane, *n*-docosane, *n*-octacosane, *n*-dotracontane, and *n*-hexatriacontane dissolved in pyridine) was added prior to trimethylsilylation. The sample set also included a quality control reference comprising *Arabidopsis thaliana* from a bulked extraction of Columbia-0 plants and a mixture of authentic metabolite standards (0.05 mg/mL). A volume of 1 µL was then injected into the GC column in a splitless mode or with a split ratio of 50:1. Spectral searching, based on the National Institute of Standards and Technology (NIST, Gaithersburg, MA, USA) algorithm incorporated in the Xcalibur^®^ data system (version 2.0.7), was done against RI libraries downloadable from the Max-Planck Institute for Plant Physiology in Golm, Germany [60] and normalized by the internal standard ribitol. Amino acids were quantified using calibration curves of standards (Sigma-Aldrich, St. Louis, MO, USA) based on 18 reference points in the range of 12.5–2200 ng. A spiking [61] method was used to distinguish between metabolites with very similar retention index and spectrum (e.g., rhamnose and fucose). Since metabolite concentrations spanned more than six orders of magnitude, a split ratio of 50:1 was implemented to estimate the levels of a few metabolites. Nevertheless, even in the split mode, the levels of sucrose and inositol were not within the linear range, and as such, their measurements may underestimate the actual change in abundance.

### 4.7. Network Analysis

The metabolic networks were built based on Spearman’s rank correlation analysis. Prior to the analysis, the relative content of each metabolite identified by GC–MS analytical platform under 35 °C was divided on its own control (mean value among 4 replicates) under 25 °C separately within each variety. The obtained ratios, reflected changes in metabolism caused by high temperature, were used for the computation of correlation matrix. The positive and negative correlation coefficient values lower than 0.5 and the coefficients that did not pass test of significance at *p* = 0.05 were filtered out from the matrix to keep only co-directed significant alterations of metabolites in the both varieties. The obtained data were transformed into Cytoscape format for network visualization (separately for each variety), where each node and edge represents metabolites and significant positive correlation between pair of metabolites, respectively. The two networks were compared and common edges were filtered out to keep only unique changes caused by high temperature in the each variety. The correlation analysis, test of significance and transformation in network format were performed using R-software, version 3.1.1 [62]. Network visualization and properties analysis were performed using Cytoscape, version 3.1.1 [63] and package “igraph” for R-software [64].

### 4.8. Statistical Analysis

Significance tests were used to test the differences in cultivar response to varied growth temperature. To avoid the effect of temporal fluctuations within the dataset and to verify the null hypothesis, all the three measurement days of the two identical experiments were combined and the average of each vine was considered as a single replicate. Data were tested for normal distribution using the Shapiro-Wilk test and for homogeneity of variance using Bartlett’s test. Tukey’s HSD test and Student’s *t*-test were performed to identify statistically significant differences between the means (*p*-value < 0.05). Principal component analysis was performed using the TMev: Multi Experiment Viewer [65] on logarithmically normalized data (base 10).

## Supplementary Materials

Supplementary materials can be found at http://www.mdpi.com/1422-0067/16/10/24276/s1.

## Abbreviations

VPD, vapor pressure deficit; *A*_N_, net carbon assimilation; *g*_s_, stomata conductance; *R*_D_, dark respiration; PPFD, Photosynthetic photon flux density; PCA, principle component analysis; NPQ, Non-photochemical quenching; ROS, reactive oxygen species; TCA, tricarboxylic acid cycle; ABA, abscisic acid; DHA, dehydroascorbate.
